# Computational Modeling in Liver Surgery

**DOI:** 10.3389/fphys.2017.00906

**Published:** 2017-11-14

**Authors:** Bruno Christ, Uta Dahmen, Karl-Heinz Herrmann, Matthias König, Jürgen R. Reichenbach, Tim Ricken, Jana Schleicher, Lars Ole Schwen, Sebastian Vlaic, Navina Waschinsky

**Affiliations:** ^1^Molecular Hepatology Lab, Clinics of Visceral, Transplantation, Thoracic and Vascular Surgery, University Hospital Leipzig, University of Leipzig, Leipzig, Germany; ^2^Experimental Transplantation Surgery, Department of General, Visceral and Vascular Surgery, University Hospital Jena, Jena, Germany; ^3^Medical Physics Group, Institute for Diagnostic and Interventional Radiology, University Hospital Jena, Friedrich Schiller University Jena, Jena, Germany; ^4^Department of Biology, Institute for Theoretical Biology, Humboldt University of Berlin, Berlin, Germany; ^5^Mechanics, Structural Analysis, and Dynamics, TU Dortmund University, Dortmund, Germany; ^6^Department of Bioinformatics, Friedrich Schiller University Jena, Jena, Germany; ^7^Fraunhofer MEVIS, Bremen, Germany; ^8^Leibniz Institute for Natural Product Research and Infection Biology, Hans Knöll Institute, Jena, Germany

**Keywords:** Liver resection, risk assessment, systems medicine, multi-scale modeling, function prediction, liver regeneration, liver metabolism, liver surgical planning

## Abstract

The need for extended liver resection is increasing due to the growing incidence of liver tumors in aging societies. Individualized surgical planning is the key for identifying the optimal resection strategy and to minimize the risk of postoperative liver failure and tumor recurrence. Current computational tools provide virtual planning of liver resection by taking into account the spatial relationship between the tumor and the hepatic vascular trees, as well as the size of the future liver remnant. However, size and function of the liver are not necessarily equivalent. Hence, determining the future liver volume might misestimate the future liver function, especially in cases of hepatic comorbidities such as hepatic steatosis. A systems medicine approach could be applied, including biological, medical, and surgical aspects, by integrating all available anatomical and functional information of the individual patient. Such an approach holds promise for better prediction of postoperative liver function and hence improved risk assessment. This review provides an overview of mathematical models related to the liver and its function and explores their potential relevance for computational liver surgery. We first summarize key facts of hepatic anatomy, physiology, and pathology relevant for hepatic surgery, followed by a description of the computational tools currently used in liver surgical planning. Then we present selected state-of-the-art computational liver models potentially useful to support liver surgery. Finally, we discuss the main challenges that will need to be addressed when developing advanced computational planning tools in the context of liver surgery.

## From systems biology via systems medicine to systems surgery of the liver

Systems biology is characterized by the application of computational models and methods to a biological question, focusing on entire biological systems and the complex interactions therein. In systems biology, an iterative cycle of model building and validation based on experimental data generation and analysis is pursued. The key purpose of computational models is the integration of biological knowledge into a mathematical representation of the underlying processes allowing *in silico* testing of new hypotheses. Systems biology applied to human diseases is an interdisciplinary approach broadening our understanding of mechanisms involved in disease development and progression. Thus, mathematical models of human diseases can enable us to discover new therapy strategies and targets.

Using the systems biology approach in a clinical setting is termed systems medicine (Wolkenhauer et al., 2013). In systems medicine, computational models are applied for disease diagnosis, prediction of disease progression, and for guidance to select suitable therapeutic strategies. In addition, computational models provide the opportunity for individualization. Patients differ in their individual anatomy, physiology, genetic background, and personal history, all of which influence the severity and course of the disease and determine the specific response of the patient. Therefore, in medicine and especially in surgery, a modeling approach is needed, which permits a patient-specific perspective on disease development and progression, taking preexisting patient-specific conditions into consideration.

Computational surgery refers to the use of computational support in the context of surgery (Garbey et al., 2012; Bass and Garbey, 2014). Computational models can guide surgery to optimize intervention and improve outcome. Such models are applied in surgery for (a) preoperative risk assessment of a patient to guide surgical planning, (b) adjustments of the procedure during a surgical intervention, e.g., by using image-based technologies, and (c) prediction of the surgical outcome accompanied by decision guiding for postoperative therapy. Computational approaches have been developed to guide surgeries for, e.g., heart failures (Kayvanpour et al., 2015; Meoli et al., 2015), brain tumors (Rockne et al., 2010; Baldock et al., 2013), and liver resections (Soler et al., 2014).

Surgical planning, especially for liver resection, benefits from computational support. The preoperative planning needs to be accurate and predictive, but also fast and easy to cope with the growing number of patients. More individualized surgical planning will be required to push the limits in liver surgery toward operating more patients with more advanced malignant tumors, higher age, and preexisting liver damage. With increasing severity of disease, the risk of postoperative liver failure rises. Here, computational support in the future will enable better risk assessment and highly individualized surgical planning for the patients requiring liver surgery, allowing to perform more successful procedures in higher-risk patients with improved outcome.

**Figure 1:**
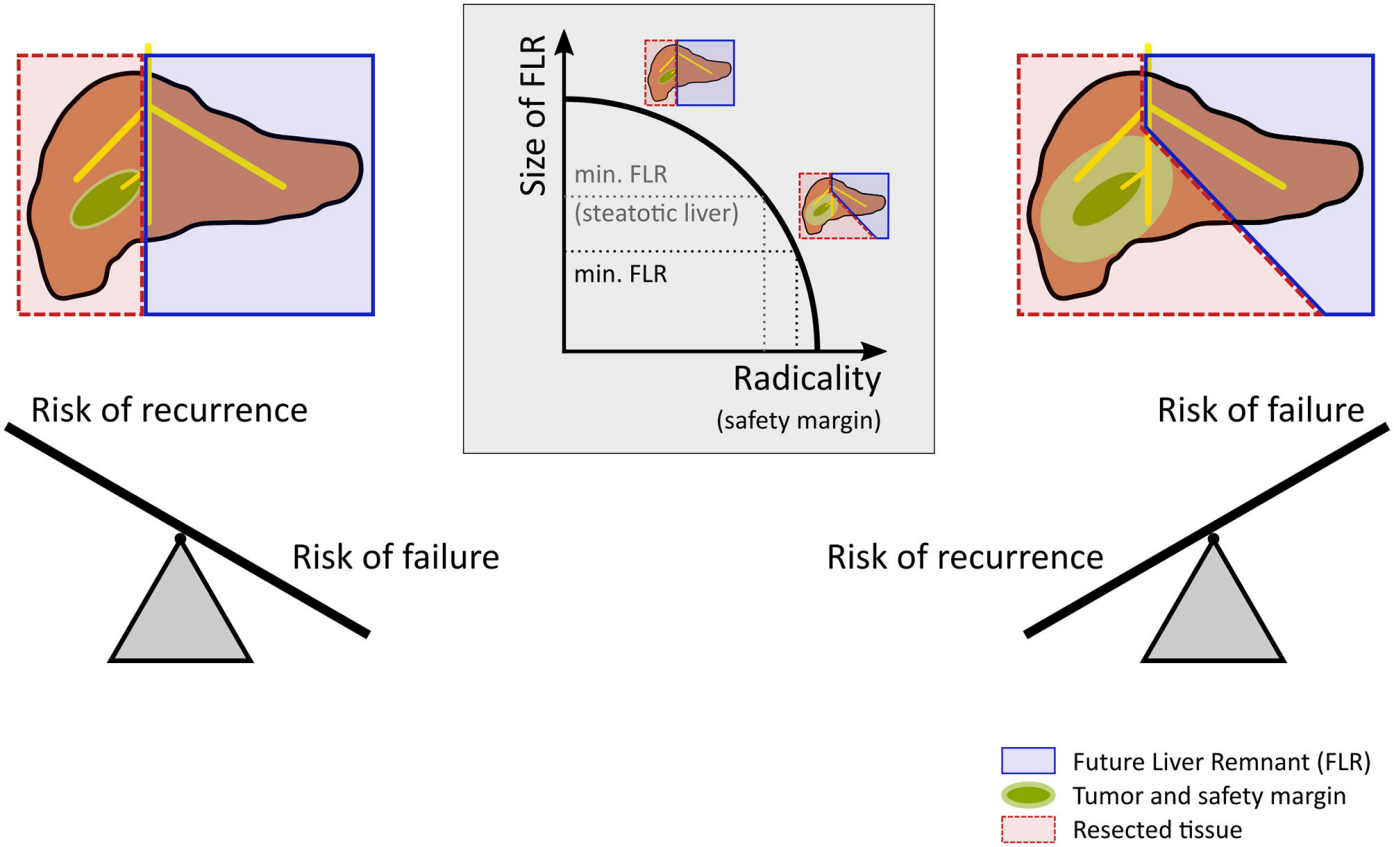
Risk assessment and decision making in hepatic resection. Planning for a safe resection of a liver tumor with a large future liver remnant (FLR) reduces the risk for postoperative liver failure but increases the risk of recurrence. In contrast, planning for an oncologic radical surgery requires a safety margin. Extending the safety margin (e.g., 10 vs. 1 mm) in case of a centrally located tumor leads to a substantially extended resection leaving a rather small future liver remnant behind, which increases the risk of postoperative liver failure. Preexisting liver disease such as steatosis increases the risk for postoperative liver failure and might therefore call for a smaller safety margin compared to livers without preexisting diseases.

Current computational support in hepatic surgery focuses on anatomical assessment. To do so, the patient's individual hepatic anatomy is taken into account to enable preoperative surgical planning. This ensures an optimal compromise between an oncologically radical resection and a remnant liver of sufficient size, see Figure 1. A radical resection involves surgically removing the tumor including a large safety margin and mitigates the risk of recurrence at the cost of an increased risk of failure. In contrast, a small safety margin maximizes the size of the liver remnant and thus reduces the risk of failure, but involves a higher risk of recurrence. Computational support of today utilizes sophisticated preoperative imaging in combination with surgical planning tools. This approach allows to assess the patient-specific anatomical condition, but does not consider the functional state of the liver. Neglecting the functional state, however, represents a serious limitation, because the success of liver surgery strongly depends on the functional quality of the remnant liver after operation, i.e., the metabolic and proliferative capacity, as well as on the adequate stress response to the surgical injury.

Future computational support must include such functional aspects. Surgical planning could be optimized by prediction of the hepatic stress response, postoperative recovery of metabolic functions, and regeneration of the future remnant liver. Both anatomical and functional assessments are needed to better predict the impact of surgical interventions. Computational support combining anatomical assessment with a risk assessment of liver (dys-)function could provide many benefits for patients undergoing liver surgery, including faster recovery, less infections, and reduced mortality, altogether leading to improved patient outcome.

Employing models from systems biology in the context of surgery, thus aiming at considering all relevant biological processes by the means of predictive computational models, is an approach that could be termed as “Systems Surgery.” Numerous computational models simulating selected hepatic functions have been developed in the field of systems biology. These models were primarily developed to improve the understanding of hepatic physiology, but their integration into current surgical planning tools is lacking so far. Extending these tools by integrating computational models involving the hepatic stress response, metabolic function, and liver regeneration would allow better prediction of the surgical risk and the postoperative course and outcome.

In this review, we provide an overview of mathematical liver modeling and its prospective application to computational liver surgery. Following a comprehensive summary of the biological and medical background relevant for liver surgery, we present an overview of state-of-the-art computational approaches supporting current liver surgical planning. Next, we provide an outline of selected liver-specific models from the field of systems biology with a special focus on their relevance for liver surgery. Finally, we identify the main challenges associated with the application of computational models in liver surgery.

## Unique challenges of liver resection

**Figure 2:**
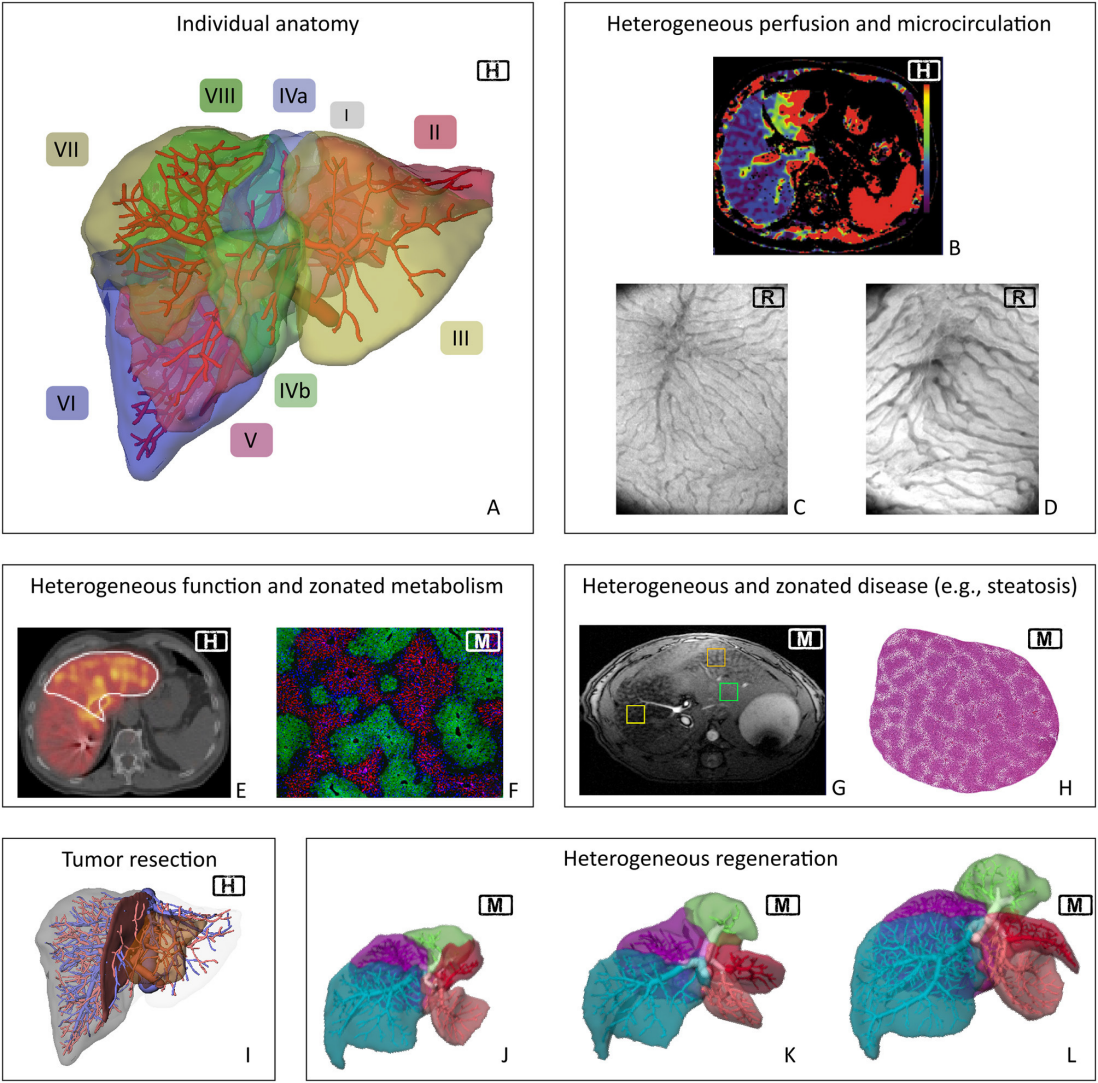
Spatial heterogeneity in liver physiology. Visualization of human individual hepatic vascular and parenchymal anatomy (**A**, the labels indicate the different Couinaud segments) is the basis of current surgical planning **(I)**. Planning currently does not take any functional heterogeneity into account. However, heterogeneity exists on the macro- and microscale in terms of hepatic perfusion (**B**, clinical perfusion CT^*^) and microcirculation [**C,D**, orthogonal polarization spectroscopy image from **(C)** normal rat liver and **(D)** rat liver after 90%PHx]. Heterogeneity also occurs in terms of regional distribution of functional activity (**E**, Mebrofenin scan of human liver^**^) and of metabolic zonation in mouse liver (**F**, periportal expression of E-cadherin and perivenous expression of CYP2E1). Furthermore, inhomogeneous distribution also occurs in case of morphologic changes due to global liver disease, here shown regional heterogeneity of fat distribution (**G**, MRT of steatotic mouse liver) as well as zonated distribution of fat accumulation in periportal hepatocytes in a mouse liver **(H)**. Current planning focuses on visualizing tumor location **(I)**. Monitoring of liver regeneration is mostly restricted to experimental or clinical studies and revealed inhomogeneous growth of the remnant lobes in mice **(J–L)**. H, human; M, mouse; R, rat. ^*^Reprinted from Cieslak et al. (2016), with permission from Elsevier. ^**^Reprinted from Wang et al. (2013), with permission from Elsevier.

The liver is a highly complex organ. It is characterized by (a) its multi-scale architecture, (b) its special perfusion system with two parallel inflows (hepatic artery and portal vein) and one outflow (hepatic vein), (c) its multitude of functions including metabolic homeostasis, synthesis of essential compounds, detoxification, and excretion of toxic substances, and (d) its high regenerative capacity after injury. Despite the seemingly regular microstructure of the liver, perfusion, functional, and regenerative capacity are distributed heterogeneously in the organ at different spatial scales, see Figure 2. Liver diseases can impair the hepatic structure, microcirculation, metabolic function, and the regenerative capacity, all potentially increasing the risk of postoperative liver failure.

### Anatomy and physiology

#### Multi-scale architecture and hepatic perfusion

The multi-scale structure of the liver consists of cells, lobules, segments, and lobes (Boyer et al., 2011). Organization of the liver in lobes and segments is based on portal supply via the two main (right and left portal vein) and eight segmental branches of the portal vein. In contrast, hepatic drainage is ensured via the three main hepatic veins (right, median, and left hepatic vein).

Hepatocytes, the main cell type of the liver, are organized in cords along the hepatic sinusoids, the capillary-like small blood vessels in the liver. This alignment of hepatocytes supports efficient functioning by (a) separating opposing pathways in spatially separated zones, (b) preventing substrate competition between different metabolic pathways, and (c) connecting consecutive pathways.

Sinusoids draining into the same central vein form the liver lobule, the functional unit of the liver on the tissue level. Perfusion of the liver lobules, also called hepatic microcirculation, is unique since the sinusoidal network receives both oxygenated blood from the hepatic artery (~20%) and (partially) deoxygenated blood from the portal vein (~80%). Alterations in the sinusoidal morphology (Figures 2C,D) lead to changes and heterogeneity in the microcirculation.

Liver lobules in the region supplied by the same segmental branch of the portal vein form one of eight segments of the liver, the so-called Couinaud segments (Couinaud, 1957), cf. Figure 2A. In contrast, each of the three main branches of the hepatic vein drains two adjacent segments (and each segment has multiple draining hepatic veins). The interplay of vascular anatomy and flow resistances at the microcirculatory (sinusoidal) level leads to heterogeneous liver perfusion (Figure 2B). This complex and highly individual anatomy makes surgical planning difficult.

#### Metabolism

The liver is crucial for maintaining metabolic homeostasis. This is achieved via synthesis, degradation, and storage of metabolites (e.g., glucose, glycogen, fatty acids, or amino acids) (Boyer et al., 2011). For instance, constant glucose levels are maintained via gluconeogenesis and glycogenolysis to continuously supply the brain and other tissues between meals (König et al., 2012). Other crucial tasks are the synthesis and excretion of bile acids, the synthesis of plasma proteins (e.g., enzymes, coagulation factors, and complement proteins), and the metabolization and detoxification of xenobiotic compounds (e.g., most drugs and toxins are cleared by the liver) (Boyer et al., 2011).

The function of individual hepatocytes depends on their position in the liver lobule, a phenomenon called metabolic zonation. Hepatocytes close to the portal field (periportal) receive oxygen-rich blood from the hepatic artery and nutrient-rich blood from the portal vein and are specialized in oxidative metabolism comprising gluconeogenesis, β-oxidation of fatty acids, and cholesterol synthesis. In contrast, hepatocytes close to the central vein (pericentral) receive lower oxygen and nutrient levels and perform glycolysis, lipogenesis, bile acid synthesis, and drug detoxification by cytochrome P450 (CYP) enzymes (Kietzmann, 2017). This zonation is mainly a consequence of differential protein expression along the sinusoid, e.g., the restricted periportal expression of E-cadherin and perivenous expression of CYP2E1 depicted in Figure 2F.

Metabolic zonation is the reason for predominantly zonal damage in response to specific challenges. For example, systemic metabolic diseases like Type 2 Diabetes mainly impact the regional specialization of periportal hepatocytes, e.g., periportal hepatocytes expressing the key gluconeogenic enzyme phosphoenolpyruvate carboxykinase (Yang et al., 2009). Similarly, initiation and progression of fibrosis during pathogenesis of liver cirrhosis affects primarily the periportal areas, since deposition of extracellular matrix originates from mesenchymal cells resident or recruited to the portal area of the liver lobule (Bataller and Brenner, 2005). In contrast, intoxication, e.g., with acetaminophen, mainly affects pericentral hepatocytes, which express the cytochrome P450 enzymes needed for metabolization of the drug (Woolbright and Jaeschke, 2017).

The metabolic functions of the liver are the result of a complex interplay between metabolism on the cellular scale, tissue structure, and perfusion of the tissue/organ. As a result of multiple heterogeneous phenomena, functional hepatocellular activity is distributed heterogeneously in the liver (Figure 2F). Consequently, important questions before liver resection are: How does a surgical intervention impact the metabolic functions of the liver? i.e., what is the remaining functional capacity of the liver for metabolic tasks after resection? Is this sufficient to support volume regeneration and functional recovery?

### Surgery and recovery

#### Resection

The incidence of liver tumors is increasing with the age of the patients. The demographic change with a constantly increasing elderly population leads to a growing number of patients in need of liver surgery (Liu et al., 2017).

Liver resection is the most common liver surgery and consists of removal of liver tissue due to focal lesions, most often malignant tumors (Abdeldayem, 2013). Malignant tumors, like hepato- or cholangiocellular carcinoma, or liver metastases, but also living liver donation, often require extended partial liver resections of more than two thirds of the liver. The extent of resection is determined by the size and location of the focal lesion and the estimated function of the future liver remnant. The function of the liver remnant depends on several factors including its volume, the size of in- or outflow compromised territories, the impairment of hepatic micro- and macro-circulation induced by resection (Nilsson et al., 2014), and the severity of any preexisting damage aggravating the microcirculatory impairment (Hossain et al., 2006).

Reduction of hepatic liver mass results in portal hypertension and portal hyperperfusion. After resection, all blood from the intestine has to pass through the reduced vascular bed resulting in an increased perfusion pressure and flow rate. Portal hyperperfusion leads to decreased arterial perfusion due to the hepatic arterial buffer response (Lautt et al., 1984). The impaired microcirculation challenges the liver remnant with a high metabolic and regenerative demand, thereby increasing the risk of liver failure.

Transecting hepatic parenchyma requires transecting branches of both the portal and the hepatic vein. Due to the anatomical disparity of two portal veins supplying, but three hepatic veins draining the liver, a certain focal in- or outflow obstruction is inevitable. The impairment of hepatic perfusion and microcirculation may cause hepatocyte dysfunction and pericentral confluent necrosis, further reducing the functional liver mass (Lee et al., 2001).

Prior to liver resection, surgeons have to assess the patient's individual risk for postoperative liver dysfunction. In case of malignant tumors, surgeons have to identify the surgical strategy best suited to allow radical oncological removal without putting the patient at risk of postoperative liver failure due to excessive removal of liver mass (Figure 1) (see also, van Dam et al., 2014; Kang and Ahn, 2017). Depending on the size, etiology, and location of the tumor, the surgeon has to define the best strategy in terms of the resection surface, but also in terms of the surgical technique such as the use of vascular occlusion to minimize blood loss. Both together determine the total parenchymal loss and the extent of damage to the remnant liver (Figure 2I). Deciding on the resection surface determines the safety margin around the tumor and the vessels which have to be transected. Therefore, a key challenge in planning liver resection is to ensure adequate vascular supply and venous drainage, both of which are essential for normal liver function. Small changes in placing the resection surface can have large effects on the size of the compromised portal/arterial inflow and venous outflow territories. In addition to the loss of liver mass by resection, compromised territories further reduce the remaining functional liver tissue, increasing the risk of the procedure.

#### Stress response

Resection causes tissue damage and induces a stress response in hepatic cells. An adequate stress response to the injury, consisting of modulation of gene expression and various signaling pathways, is imperative for the patient's survival and recovery. Particularly, the impairment of hepatic microcirculation after resection, which is accompanied by an altered substrate delivery via blood to the hepatocytes (Siu et al., 2014; Dold et al., 2015), makes an adaptation of the metabolic activity necessary. Here, a sufficient supply with oxygen for oxidative processes is required, but local hypoxia caused by the impaired perfusion leads to an increased production of reactive oxygen species (ROS) upon reperfusion (Bhogal et al., 2010). Physiologically, ROS are signaling molecules involved in mediating an adequate stress response to tissue injury by modulating metabolic adaptations and activating the innate immune system. Pathophysiologically, however, excess ROS may cause cell damage. Particularly, if vascular exclusion is used during liver resection to minimize blood loss (Garcea et al., 2006), the level of ROS production raises, ultimately resulting in vast cell damage, decreased metabolic function, and ischemia/reperfusion injury (Zhang et al., 2007). This hampers the function of the remnant liver, again contributing to the risk for postoperative liver failure. Subsequently, the surgeon is faced with a critical trade-off between the advantage of reduced blood loss and the risk of ischemia/reperfusion injury (van Riel et al., 2016).

The hepatic stress response also triggers, besides metabolic adaptations, an activation of the regenerative process (Michalopoulos, 2017) and a local inflammatory response (Alazawi et al., 2016). The latter is not only important for removal of damaged and necrotic cells and triggering regeneration, but also to prevent infections. After surgery, patients are faced with increased risk for complications, such as focal infections, the systemic inflammatory response syndrome, or sepsis (Alazawi et al., 2016). This risk increases with postoperative hepatic dysfunction, which is ultimately determined by the remnant liver volume (Schindl et al., 2005). The levels of inflammatory cytokines, such as IL-6, IL-8, and MCP-1 (monocyte chemotactic protein-1) correlate with the degree of tissue damage and reflect the early response to surgical injury (Badia et al., 1998; Strey et al., 2011; Friedman et al., 2012).

#### Regeneration

The liver possesses a high regenerative capacity (Fausto et al., 2012). This unique capability ensures restoration of size and function after surgical, physical, or chemical injury (Figures 2J,K,L). In principle, two different types of damage require restoration of the liver mass: (a) cell death due to systemic injury of the liver, predominantly occurring in a zonated manner, and (b) tissue loss due to removal of liver segments or lobes via resection.

Original liver mass after resection is restored by mature hepatocytes in the residual liver undergoing oscillating cell divisions (Miyaoka and Miyajima, 2013). The first wave of division encompasses about 60% of the hepatocytes, followed by waves of considerably less proliferation (Zou et al., 2012; Miyaoka and Miyajima, 2013). The immediate regenerative response after resection is mediated by HGF and IL-6, the so-called priming factors of liver regeneration allowing hepatocytes to re-enter the cell cycle (Fausto and Campbell, 2003). As part of the stress response of liver cells to tissue injury, the process of liver growth is highly controlled by a variety of signaling molecules involving, among others, cytokines, growth factors (Böhm et al., 2010), and hormones (Marino et al., 1992).

Substantial recovery of the liver mass occurs within 10 days, and 80 to 90% of the original liver mass is reached within 6–12 months following 70% resection (Nadalin et al., 2004; Kele et al., 2012). In contrast, reports about the recovery of liver function are highly variable, as this depends on the specific aspect under investigation. For instance, liver biochemical parameters [bilirubin, international normalized ratio (indicator of blood coagulation)] return to normal within 10 days, whereas cholinesterase, albumin, and galactose elimination capacity recover within 90 days (Nadalin et al., 2004).

The liver accumulates lipids during regeneration (Michalopoulos, 2007; Zou et al., 2012; Miyaoka and Miyajima, 2013). These lipids derive from an increased adipose tissue lipolysis and provide energy substrates for the proliferation of hepatocytes in the liver (Farrell, 2004; Fausto, 2004; Walldorf et al., 2010). While this “physiological” post-resection steatosis is beneficial, excess lipid accumulation in hepatocytes causes hepatocyte death and impaired liver regeneration. This is of special interest after extended liver resections, because a small liver remnant has lower lipid storage capacity, and thus a higher risk of lipid overload and organ dysfunction, than a larger remnant. Since obviously the liver is unable to regulate the amount of lipid uptake in relation to its size after resection, extended resections lead to a pathophysiological shift from utilization during regeneration to excess storage (Shteyer et al., 2004; Hamano et al., 2014; Tautenhahn et al., 2016).

Ultimately, the course of liver regeneration depends on the functional capacity of hepatocytes in the liver remnant. The loss of liver tissue puts an additional stress on the residual parenchyma to take over the metabolic tasks previously accomplished by the whole liver prior to resection. This is critical in situations where hepatocyte function is already impaired by preexisting damage, like, e.g., hepatic steatosis as discussed below.

### Preexisting diseases

Preexisting global liver diseases can increase the risk of liver surgery. Liver diseases affecting the whole organ comprise metabolic, inflammatory and autoimmune, or infectious diseases. Such diseases compromise architecture, function, and regeneration of the liver and are often associated with or may lead to steatosis, cholestasis, and fibrosis. In the following, we focus on hepatic steatosis to delineate how one exemplary liver disease may aggravate liver surgery.

Hepatic steatosis is defined as an excessive accumulation of fat in the hepatocytes. Steatosis starts with development of small droplets (microvesicular steatosis) progressing to large droplet formation (macrovesicular steatosis). Depending on the etiology, fat accumulation often starts in one specific zone, e.g., in the pericentral zone in case of ethanol-induced toxic etiology. Besides zonal accentuation (Figure 2H), fat distribution can also be subject to regional variations, resulting in substantial heterogeneity in the regional fat content (Figure 2G; Capitan et al., 2012; Idilman et al., 2016; Schwen et al., 2016).

Patients with steatosis have a higher surgical risk than patients without steatosis (Kooby et al., 2003; Clavien et al., 2007; McCormack et al., 2007). Several reasons contribute to the risk: (a) Steatosis causes an alteration of hepatic architecture leading to an inhomogeneous impairment of perfusion and to an increase in portal pressure (Seifalian et al., 1998). Impaired perfusion is at least partially caused by swollen fatty hepatocytes and sinusoidal “capillarization” (Brock and Dorman, 2007) and reduces oxygen and nutrient supply, contributing to the impaired regenerative response (Yarbrough et al., 1991). (b) Steatosis induces metabolic impairment, which aggravates post-resection lipid overload. Preexisting steatosis is the result of the pathologic shift of lipid metabolism from utilization to storage due to regulatory impairment. This impairment is not resolved after PHx. Therefore, fat further accumulates instead of is being utilized for regeneration. This extends lipotoxic exposure for each single hepatocyte, thus augmenting cell death by, e.g., ROS as described below. Hence, preexisting steatosis exacerbates the reduction of the functional capacity of the liver after resection. (c) Steatosis aggravates hepatic ischemia/reperfusion injury. The increased metabolic supply and the impaired microcirculation in the fatty liver “disrupt hepatic oxygen homeostasis,” ultimately leading to local tissue hypoxia (Suzuki et al., 2014). This preoperative condition makes fat-loaded hepatocytes particularly vulnerable to ischemia/reperfusion due to an increased level of oxidative stress. Thus, aberrant lipid accumulation in hepatocytes sensitizes them against ischemia/reperfusion injury, which occurs during the surgical procedure of partial liver resection and transplantations (El-Badry et al., 2011; Kimura et al., 2016).

Taken together, flow restrictions due to excessive lipid accumulation, hepatocyte impairment of lipid metabolism in association with oxidative stress, and cell death impair liver regeneration after resection in case of preexisting fatty liver diseases. This is corroborated by clinical and experimental studies indicating that preoperative metabolic interventions improve the impaired regenerative response of the steatotic liver (Liu et al., 2013). In mice fed with a high fat diet, which induced hepatic steatosis, omega-3 polyunsaturated fatty acids given 1 h prior to operation, ameliorated liver regeneration after both two thirds and 86% partial liver resection by attenuating hepatic steatosis and ischemia/reperfusion injury (Linecker et al., 2017).

In summary, preexisting liver diseases such as hepatic steatosis increase the surgical risk for liver resection in multiple aspects. Currently, this multi-dimensional risk is difficult to quantify preoperatively for the individual patient. Therefore, tools are needed to promote an integrated risk-assessment based on different assessment modalities taking as many aspects as possible into consideration.

## Computational-aided surgery for liver resection

**Figure 3:**
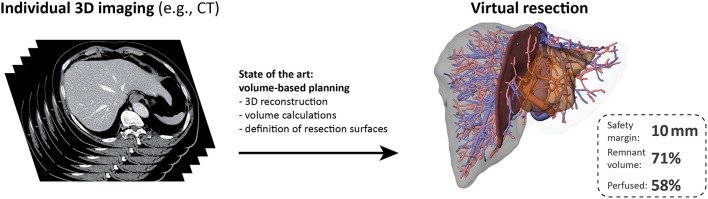
Preoperative surgical planning of today. Current surgical planning tools allow visualization of the individual liver volumes, hepatic vascular anatomy and the corresponding portal venous and hepatic venous territories. Interactive tools allow to perform virtual liver resections and the (perfused) volume of the future liver remnant can be calculated for the selected resection surface. The resection surface can be modified according to the width of the safety margin. The state of the art of surgical planning for liver resection is based on the assumption that all liver volume is functionally equal without any heterogeneity. Such an approach does not take functional aspects into account. The stack of CT images on the left was adapted from (Figure 1B in Chung et al., 2013), image license: CC-BY (https://creativecommons.org/licenses/by/3.0/).

Current computational tools primarily support surgical planning and intraoperative guidance based on images of the individual patient anatomy, but do not include functional aspects (see Figure 3). Surgical planning needs to address questions (Hansen et al., 2014) related to (a) anatomic resectability, (b) safety margin widths around lesions, and (c) resection strategy, but also to (d) the functional capacity of the future remnant liver.

### Medical imaging techniques for liver surgery

A variety of imaging techniques is available for the detection and differential diagnosis of liver pathologies, the assessment of liver anatomy, and more lately also for the spatially resolved evaluation of liver function. The armamentarium includes ultrasonography, computed tomography (CT) and magnetic resonance imaging (MRI) as well as nuclear medical imaging modalities. The latter, for instance, play an important role in detecting microvascular invasion of carcinoma preoperatively using ^18^F fluorodeoxyglucose (FDG) PET-CT (Kobayashi et al., 2016), but also allow to assess hepatic perfusion and excretory function based on hepatobiliary sequence scintigraphy (Cieslak et al., 2015, 2016) using different tracers, such as ^99m^Tc (technetium), ^99m^Tc-galactosyl, or ^99m^Tc-mebrofenin.

CT is a core technology for tumor staging and volumetric evaluation of the liver. It enables precise visualization of the tumor location with respect to the intrahepatic vascular anatomy. In fact, the first computational planning tools considering the individual hepatic anatomy were developed on the basis of CT imaging (Radtke et al., 2007; Lehmann et al., 2008). Currently, CT is the most common first-line imaging modality for staging and monitoring of liver diseases (Pinato et al., 2017) as well as postoperative risk prediction based on future remnant liver volume (Vauthey et al., 2002; Truant et al., 2007). Advantages of CT include low cost, high availability, and fast scan times. With perfusion CT, functional assessment of the liver is made possible by performing dynamic CT acquisitions following intravenous administration of contrast agent to extract blood supply characteristics into the tissue (Wang et al., 2013).

More recently, liver ultrasonography (US) and MRI have gained ground with regard to their use in the detection, characterization, and assessment of the response to treatment of focal and diffuse liver diseases (van Beers et al., 2015).

Ultrasonography allows early diagnosis, treatment management, and monitoring therapy outcome (Matos et al., 2015). Recent developments in dynamic contrast-enhanced US (Lencioni et al., 2007) and US-based elastography (Serai et al., 2017; Wang et al., 2017) have facilitated dedicated and specific liver pathology assessment. Contrast-enhanced US promises great potential to evaluate tumor vascularization in real time (Rübenthaler et al., 2017b) and has meanwhile evolved to a minimally invasive imaging modality for evaluating unclear liver lesions (Bartolotta et al., 2016; Rübenthaler et al., 2017a). However, there are still several open issues concerning standardization, operator dependency, 3D capabilities, and the potential for quantitative perfusion. US-based elastography allows predicting postoperative liver failure based on the elasticity of the tissue (Shen et al., 2017).

MRI stands out for its superior soft tissue contrast and the absence of ionizing radiation. MRI makes it possible to evaluate different tissue properties, including fat content, restriction of water diffusion, or increased T_2_-relaxation times, all of which support lesion detection. Furthermore, in combination with a liver-specific contrast agent such as gadoxetic acid (Gd-EOB-DTPA), monitoring the perfusion dynamics and the uptake of the agent allows functional assessment of the liver (Imbriaco et al., 2017; Szklaruk et al., 2017; Zhou et al., 2017), thereby improving the detection of liver carcinoma and classification of microvascular invasion in hepatocellular carcinoma. Thus, MRI is a versatile modality for creating detailed, anatomically accurate models for computationally aided liver surgery (Oshiro and Ohkohchi, 2017; Rutkowski et al., 2017). In addition, it offers further potential in form of magnetic resonance cholangiography or contrast enhanced magnetic resonance angiography allowing comprehensive assessment of a patient's biliary and vascular status and possible complications (Boraschi et al., 2008).

Localized magnetic resonance spectroscopy is a non-invasive method to quantify the relative fat fractions of liver tissue, thus providing an elegant means to assess preexisting steatosis (Chiang et al., 2016; Di Martino et al., 2016; Krishan et al., 2016; Kramer et al., 2017). It is often used as gold standard for determining the proton density fat fraction with the potential to replace liver biopsy and takes advantage of the so-called chemical shift, which is based on magnetic field shielding by the molecules' electrons. The different chemical shifts between hydrogen bound to water and lipids can also be utilized by fat-water quantification imaging sequences (Hedderich et al., 2017; Jhaveri et al., 2017), which offer more detailed insight into the spatially inhomogeneous distribution of fat deposits in a steatotic liver (Jang et al., 2017). This way, image-based MR methods may overcome some of the limitations of magnetic resonance spectroscopy associated with restricted spatial coverage and subjective positioning of the volume of interest, which may adversely affect accuracy.

As mentioned before, nuclear medicine also offers very specific imaging methods to support liver surgery. Using the radio-fluorinated carbohydrate (Mun, 2013) 2-[(18)F]fluoro-2-deoxy-D-galactose and PET-CT detection to assess galactose clearance, improved detection of hepatocellular carcinoma has been demonstrated (Horsager et al., 2016). For patients undergoing a major resection, risk assessment and prediction of remnant and future liver function based on hepatobiliary scintigraphy using ^99m^Tc-mebrofenin has been shown to provide better sensitivity, specificity, and positive/negative prediction values compared to conventional remnant liver volume-based risk assessments (de Graaf et al., 2010; Cieslak et al., 2016). Though this method is currently used only in explorative studies at a small number of sites, combining ^99m^Tc scintigraphy with the liver-specific functionalization agent mebrofenin appears fairly promising for spatially resolved, accurate functional assessment of the liver.

Taken together, a diversity of imaging modalities and methods is currently available which, however, are not evenly spread and readily available at all centers for daily routine yet. While basic CT, US, and MRI are ubiquitously performed, particularly the more recently developed methods in magnetic resonance imaging and spectroscopy, contrast-enhanced US and nuclear medicine, despite being very promising, are so far largely limited to specialized centers.

### Current virtual resection tools

Presently, most computational models supporting liver resection planning are based on individual patient anatomy (see Figure 3), in particular the spatial relationship between tumor location and hepatic vascular systems (e.g., Fishman et al., 1996; Marescaux et al., 1998; Lang et al., 2005). Accurate visualization of this spatial relationship is important for the surgical success of a liver resection (Saito et al., 2005), and can be achieved by 3D imaging and appropriate visualization techniques (e.g., Fishman et al., 1996).

More advanced approaches support the planning of the resection by virtual resection tools. HepaVision (now MeVis LiverAnalyzer; Schenk et al., 1999) and LiverPlanner (Reitinger et al., 2006) provide a patient-specific resection planning proposal and highlight different safety margins sizes and affected vascular structures as well as the remaining total and perfused liver volume. Thus, the surgeon can adjust the desired safety margin, which influences the resection proposal. Such planning software is implemented in clinical routine for extended liver resection planning.

Recent developments integrate additional biophysical properties of the liver. Liversim (Oshiro et al., 2015) is a novel virtual hepatectomy simulation software tool, which additionally captures motion and deformation of the liver caused by the intervention. A soft-tissue deformation model including hyperelasticity, porosity, and viscosity of hepatic tissue allows simulating realistic liver deformations and intrahepatic displacements in real time for surgery training (Marchesseau et al., 2010) and planning. Modern medical imaging coupled with computational fluid dynamics (CFD) modeling also facilitates predicting patient-specific alterations in hepatic hemodynamics in response to partial hepatectomy (Rutkowski et al., 2017).

### Volume-based risk assessment in clinical routine

Optimizing the surgical planning phase by computer-assisted risk analysis can enhance surgery success. In case of hepatic cancer, liver resections can be supported by a preoperative, computer-based calculation of the remnant liver volume (Lang et al., 2005). Hepatic volume estimation by a surgical planning software tool revealed enhanced accuracy compared to the radiologist's volume estimations based on planimetry of a single CT/MR slice (DuBray et al., 2011). The ratio of pre- and postoperative liver tissue volumes, as a rough approximation of postoperative liver function, has been included in virtual surgical planning systems (e.g., Glombitza et al., 1999a,b; Simpson et al., 2014; Hallet et al., 2015; Oshiro and Ohkohchi, 2017).

The aim of liver tumor resection is the complete removal of the cancer. The surgical planning phase encompasses the determination of an optimal safety margin width around the tumor locations (Vandeweyer et al., 2009). Here, a trade-off exists between adequate remnant liver function and sufficient safety margin width. Some computer-based resection planning tools that link visualization of liver structures with an additional volume-margin function support precise operation planning (Glombitza et al., 1999b; Preim et al., 2002; Hansen et al., 2009), thereby enhancing the awareness of the surgical risk and supporting the decision for a smaller resection volume compared to surgical planning based only on conventional 2D/3D viewer application (Hansen et al., 2014).

### The challenge of function-based risk assessment

Current surgical planning tools focus on the estimation of liver volume as a surrogate predictor of remnant liver function. The underlying assumption is that all hepatocytes contribute equally to liver function. This, however, neglects the spatial heterogeneity of liver metabolism and perfusion, potential alterations of hepatic function in the presence of a liver disease, or individual variations in metabolic function due to genetic variants, or as a consequence of lifestyle.

Consequently, accurate assessment of the preoperative risk requires improved evaluation of the individual functional capacity and prediction of this capacity for the future liver remnant. Such an improved assessment is essential for the ultimate goal of prevention and early detection of postoperative liver failure (Daylami et al., 2016). The measured changes in metabolic function associated with liver surgery and disease depends on the substance used in the function test. However, as outlined above, the liver is a multifunctional organ, for which a single functional assay only provides information about one specific aspect of hepatic function.

Only few diagnostic tools are currently available for measuring metabolic function of the liver. Information about the metabolic functional capacity can be obtained by means of dynamic quantitative liver function tests, which measure the clearance of selected substances specifically metabolized by the liver such as, e.g., the clearance of caffeine (Fuhr et al., 1996), indocyanine green (De Gasperi et al., 2016), or methacetin (LiMAx test, Jara et al., 2015). The metabolic clearance of a selected compound is hereby used to approximate global metabolic liver function as a cumulative effect. Hence, it is necessary to understand the underlying metabolism of the relevant substances and its alteration due to disease and surgery.

This approach cannot provide information about spatial heterogeneity such as, e.g., inhomogeneously distributed steatosis throughout the organ resulting in areas with higher and lower functional activity. Furthermore, such approaches cannot discriminate between the influences of cellular metabolic activity and altered perfusion or liver size after surgery. Here, novel methods are needed to accurately reflect severity, distribution, and composition of fat accumulation and, even more importantly, the resulting spatially resolved functional impairment.

A comprehensive function-based risk assessment requires consideration of all relevant clinical information. Such an assessment needs to integrate information about resection volume/amount, preoperative metabolic impairment in case of preexisting liver disease, intraoperative damage to the future liver remnant as well as metabolic and regenerative capacity of the future liver remnant. To achieve this, multi-scale computational approaches are needed for integrating all relevant processes into one comprehensive risk prediction. Currently, however, only some of the required features are already available (see section below on “Computational Modeling of Liver Diseases Relevant for Surgeries”), but not within one comprehensive risk assessment tool.

One first attempt to extend surgical planning beyond mere visualization and volume estimation has been provided recently by a model, which simulates postoperative liver regeneration in a patient-specific manner (Yamamoto et al., 2016). This model provides predictions of the duration of the postoperative recovery period and possible complications.

## Computational liver models relevant for liver surgeries

Regulation and maintenance of liver function involves complex biological processes spanning multiple spatial and temporal scales. Spatial scales range from the intracellular level up to the level of the organism, whereas temporal scales have to reflect time periods of seconds to years (e.g., metabolism in seconds to days, regeneration over weeks, or disease progression over months). Various biological processes play a role for hepatic function in liver surgery, particularly important are the hepatic stress response, metabolic adaptations, and regeneration.

Thus, multi-scale-oriented modeling approaches are especially suited to provide a more comprehensive understanding of hepatic processes and mechanisms. Multi-scale-oriented modeling consists of developing “simple” separate models of certain sub-aspects or scales of the function of interest. Subsequent model integration links input and output variables of these separate models and leads to a more comprehensive combined model, possibly spanning multiple scales. This so-called hierarchical modeling approach (Cedersund and Strålfors, 2009; Nyman et al., 2011) allows adapting the model resolution to the corresponding research question (Kirschner et al., 2014). Current computational models can simulate a variety of selected liver functions, see Tables 1–3 and reviews (Bogle et al., 2012; Hetherington et al., 2012; Sumner et al., 2012; Fisher et al., 2014; Petta et al., 2016).

**Table 1:** Selection of existing computational models to address the stress response with potential relevance for surgical planning, sorted according to spatial scale (*cell to organism*).

**Scale**	**Modeling Approaches**
Cell	*Intracellular signaling to adjust hepatic function to external conditions, e.g.*, Dietary composition—**ODE** (Woller et al., 2016) Reactive oxygen species production—**ODE** (Selivanov et al., 2009, 2012; Gauthier et al., 2013; Smith and Shanley, 2013; Markevich and Hoek, 2015) Hepatocyte growth factor network—**ODE** (D'Alessandro et al., 2015b)
	*Local inflammatory reaction due to tissue damage (i.e., activation of the immune response)* IL-1 and IL-6 signaling network—**Boolean network** (Ryll et al., 2011) Hepatic stellate cell activation (signaling)—**PetriNet** (Kuttippurathu et al., 2014)
Lobule	*Inter- and intracellular interactions* To trigger liver regeneration—**ODE** (Cook et al., 2015) Involved in signal propagation—**ODE** (Verma et al., 2016)
	*Establishment of zonation patterns* Wnt/ß signaling—**ODE** (Kogan et al., 2012; Benary et al., 2013) Hedgehog signaling— **fuzzy-logic-based** (Schmidt-Heck et al., 2015)
Organ	*Simulating patient's immune response (immune cells, blood concentrations of various signal molecules, blood pressure, tissue damage)* To pathogen infection—**ODE** (Clermont et al., 2004) (*in silico* clinical trials to predict outcome of sepsis) To surgical trauma and hemorrhagic shock—**ODE** (Chow et al., 2005; Lagoa et al., 2006)
Organism	(none)
Multi-Scale Integration	(none)

*ODE, Ordinary differential equations*.

The following sections present selected models/modeling approaches for addressing liver functions, which might be essential for future multi-scale models supporting liver resection: (a) the hepatic stress response following physical damage, (b) the metabolic pathways affected by surgery, as well as (c) the regeneration of liver volume and function recovery.

### Stress response induced by physical damage

Resection induces a hepatic stress response, which involves a modulation of signaling pathways and gene expressions. Understanding the signaling network of the liver and how the signaling affects metabolism, inflammatory processes, and regeneration is important to assess the overall hepatic stress response to resection. Signaling pathways are interconnected in a non-linear fashion, involving complex interactions as well as feedforward and feedback loops (D'Alessandro et al., 2015a). An intuitive understanding of the signaling network is impossible due to this intricate dynamic behavior. Here, mathematical modeling can be used to disentangle the complex crosstalk between signaling pathways. Based on this knowledge, further mathematical models can be developed, which connect the degree of surgical injury with liver function, inflammatory response, and regenerative capacity. Such models enable predictions of the hepatic response to surgical intervention and possible postoperative complications in regard to an impaired metabolism or regeneration based on the degree and/or location of surgical damage. Here, understanding the relation between remnant liver volume, hepatic metabolic function, and the local immune response is important to optimize liver resections planning (Schindl et al., 2005).

In the following, we provide a short overview of existing computational models of hepatic signaling pathways to illustrate the current state of knowledge. Then, we focus on ROS as important signaling molecules (Dickinson and Chang, 2011; Ray et al., 2012) and as a source of cellular damage impairing hepatic metabolism and activating inflammatory processes after surgical injury. Finally, we take a closer look at current models considering the inflammatory response and the activation of the innate immune system. A summary of selected models available to address the hepatic stress response, which might be relevant for surgical planning, is given in Table 1.

#### Models of signaling pathways

A variety of mathematical models of hepatic signaling processes were developed, mostly using ordinary differential equations (ODEs). Aspects covered by such models include, e.g., the origin of zonation patterning (e.g., Wnt/β-catenin signaling pathway, Kogan et al., 2012; Benary et al., 2013), the propagation of calcium waves at the lobular scale involved in the regulation of diverse hepatic functions (Verma et al., 2016), or the link between the circadian clock and hepatic metabolism (Woller et al., 2016). These models elucidate important features in the regulation and signaling of hepatic function. One example is a fuzzy-logic based model of the GLI-code, the set of three transcription factors linking hedgehog signaling with regulation of metabolic zonation as well as lipid and drug metabolism in hepatocytes (Schmidt-Heck et al., 2015). This relation was also used to explain the link between hedgehog signaling and steatosis (Matz-Soja et al., 2016).

Mathematical models of signaling pathways relevant for liver surgery are necessary to predict, how the liver responds to interventions. One promising approach is the hybrid modeling strategy (D'Alessandro et al., 2015b), which links interaction graph modeling of the signaling network with ODEs, thus permitting time-dependent simulations. In a first step, the minimal model structure of a signaling network is identified by interaction graphs. Then, subsequent analysis of ODE models of this minimal model structure allows the identification of the best model version. Such a modeling strategy helps to disentangle the intracellular signaling network structure and to predict the outcome of disturbances. The strategy was applied to the hepatocyte growth factor-induced signaling network and allows the prediction of the network response to interventions. An accurate and precise prediction of the response of a relevant signaling network to liver resection would allow better assessment of, e.g., course of regeneration, and thus help to optimize surgical procedures or even to decide for or against an operation.

#### Models of reactive oxygen species

Reactive oxygen species play a prominent role in the signaling network being active after liver resection, and influence, for example, the JNK pathway (Seki et al., 2012). During the first hours after liver resection, an increased level of ROS was observed (Guerrieri et al., 1999; Lee et al., 1999). This high ROS level is involved in the initiation of regenerative (Fausto, 2000; Tormos et al., 2013) and inflammatory processes (Bhogal et al., 2010; Seki et al., 2012) in response to the injury. Moreover, these oxygen-based radicals are toxic and lead to oxidative stress, which can result in vast cell damage and decreased metabolic function.

Therefore, computational models focusing on ROS linked to relevant signaling pathways may be helpful in understanding (and predicting) the hepatic surgical stress response. Based on ODEs, several computational models have considered various aspects of the production and degradation of ROS (e.g., Selivanov et al., 2009, 2012; Gauthier et al., 2013; Markevich and Hoek, 2015). Furthermore, a mathematical model simulating the complex regulation of insulin signaling by ROS yielded insights into both protective and detrimental effects of ROS (Smith and Shanley, 2013). The comprehensive overview by Pereira et al. (2016) of the intracellular ROS crosstalk, including the previous models, provides a systems-level examination of the complexities of ROS as intracellular signal molecule and toxic compound. However, mathematical models describing ROS signaling pathways relevant for liver surgery are still missing and no specific model of the processes leading to ischemia/reperfusion injury in the liver exists.

#### Models of inflammation and the immune response

The stress response of the liver involves also a local inflammatory reaction. The signaling process starts with the release of so-called damage-associated molecular patterns (Zhang et al., 2010) from stressed hepatocytes. These signals activate the production of pro-inflammatory cytokines in Kupffer cells, which initiate the recruitment of leukocyte subsets to the injured site (van Golen et al., 2012). Immediately after surgery, the concentration of cytokines provides some hint of the degree of tissue damage (Badia et al., 1998; Strey et al., 2011; Friedman et al., 2012). Genome-wide gene expression measures were used to fit and refine a literature-based Boolean model of interleukin 1 and interleukin 6 signaling as a representation of hepatocellular inflammation and proliferation (Ryll et al., 2011). Novel relations between proliferation-associated processes were identified in this study, which provided better understanding of the stress response after surgery. In addition, the release of interleukin 6 and tumor necrosis factor alpha by activated Kupffer cells triggered the cell cycle entry of hepatocytes and therefore initiates liver regeneration (van Mierlo et al., 2016). An ODE model to simulate the cytokine signaling and the increased metabolic demand as triggers for regeneration has been established (Cook et al., 2015). Depending on signaling patterns, the model showed the existence of different modes of regeneration after resection and emphasized the importance of Kupffer cell cytokine signaling for the regenerative process.

Computational models can help to elucidate important links between hepatic function and the immune response. Postoperative hepatic dysfunction augments the probability to acquire an infection (Schindl et al., 2005). Thus, quantifying the relationship between liver volume, hepatic function, and the immune response is of major importance to enhance the safety of liver resections (Schindl et al., 2005). For example, the Petri net approach was used to clarify the timing and regulation of activation of hepatic stellate cells (Kuttippurathu et al., 2014), an important cell type for the modulation of the innate immune response. Relevant signaling pathways, such as NF-κB and STAT3, were coupled to the regulation of microRNAs and the model elucidated the driving regulatory factors in the process of stellate cell activation. Another modeling framework used a set of ODEs to simulate key inflammatory processes (see Clermont et al., 2004; Chow et al., 2005 for model details) initiated by surgical trauma and hemorrhagic shock to predict global damage and dysfunction as an approximation to patient survival (Lagoa et al., 2006).

#### Perspective: stress response models in computational liver surgery

In conclusion, computational models coupling signaling and the innate immune response already exist. Their usage has greatly improved the understanding of the immediate hepatic stress response to physical damage. However, mathematical models linking, for example, the postoperative metabolic impairment with ROS-induced cellular damage are still missing. The cell damage caused by an increased level of ROS after an operation affects the function of the remnant liver and, therefore, is relevant for the risk assessment of postoperative liver failure. Future computer-based predictions of the remnant liver function should take into account the preoperative metabolic capacity of the liver as well as the possible postoperative impairment caused by oxidative stress. Also, computationally supported identification of patients at specific risks for developing sepsis or acquiring a serious infection after the intervention is still lacking.

The challenge for modelers in the field of hepatic signaling is now to shift the focus to a surgical perspective. Computational models are needed that incorporate the knowledge of signaling networks and the hepatic stress response, thus linking the degree of surgically caused tissue damage to impairments in metabolism and to the activation of the inflammatory response. This would enable a more precise computer-supported risk assessment before resection. It is conceivable that such a tool predicts the surgical outcome in response to the expected surgical tissue damage and guides the decision of the surgeon for or against a resection and for postoperative therapy strategy.

### Metabolism

Removal of functional liver tissue exceeding a critical cut-off leads to a compromised metabolic liver function and ultimately to liver failure. For accurate and quantitative evaluation of the remnant functional capacity, the metabolic function of the remaining volume must be determined. This function depends on alterations of metabolism, perfusion, and morphology in the acute phase after surgical intervention and during regeneration. Computational models of hepatic metabolism can provide a better understanding of the functional capacity of the healthy liver (for an overview see also Cvitanović et al., 2017) and the metabolic alterations occurring with disease, after liver resection, and during regeneration.

In the following, we provide an overview on computational models describing metabolic liver function with a special focus on models incorporating multiple scales and coupling liver morphology and perfusion to metabolism, followed by an outlook on the application of such models to liver surgery. A summary of selected models available to address the hepatic metabolism, which might be relevant for surgical planning, is given in Table 2.

**Table 2:** Selection of existing computational models addressing metabolism with potential relevance for surgical planning, sorted according to spatial scale (*cell to organism*).

**Scale**	**Modeling Approaches**
Cell	*Metabolization of drugs* • Toxicity and timescale analysis, acetaminophen detoxification—**ODE** (Reddyhoff et al., 2015; Sluka et al., 2016)
	*Glucose metabolism* • Glucose homeostasis and hormonal regulation—**ODE** (König and Holzhütter, 2012; König et al., 2012)
	*Lipid metabolism—***ODE** • Steatosis development (Schleicher et al., 2014) • Insulin resistance & high intake diets (Ashworth W. et al., 2016) • Beta-oxidation (van Eunen et al., 2013)
	*Genome scale metabolism*—**FBA** • Flux predictions under various conditions (Gille et al., 2010; Jerby et al., 2010; Agren et al., 2014; Naik et al., 2014) • Gain and loss of enzymes (Pagliarini et al., 2016) • Integration of transcriptomics & metabolic data (Hyötyläinen et al., 2016) • Alterations of pathways in NAFLD (Mardinoglu et al., 2014)
Lobule	*Perfusion* • Resolved hepatic microvascular system—**PDE** (Rani et al., 2006) • Anisotropic permeability—**multiphase-PDE** (Ricken et al., 2010, 2013) • Role of vascular septa—**PDE** (Debbaut et al., 2014) • Multilevel approach CFD—**PDE** (Peeters et al., 2015) • CFD boundary conditions—**PDE** (Aramburu et al., 2016)
	*Perfusion + glucose metabolism* • Glycogen patterns & zonation—**multiphase-PDE** + **ODE** (Ricken et al., 2015) • Zonated glucose metabolism—**ODE** (Chalhoub et al., 2007; Ashworth W. B. et al., 2016)
	*Perfusion + lipid metabolism* • Zonated lipid metabolism—**ODE** (Schleicher et al., 2014) • Zonated damage & steatosis—**ODE** (Ashworth W. et al., 2016)
	*Perfusion + drug clearance* • Sinusoidal unit/representative sinusoid—**PDE**+**ODE** (Schwen et al., 2015)
	*Perfusion + ammonia detoxification* • CCl4 damage—**AB**+**ODE** (Schliess et al., 2014; Ghallab et al., 2016)
Organ	*Perfusion + Metabolization* • Well-stirred compartments for acetaminophen detoxification (Reddyhoff et al., 2015)—**ODE** • Spatially resolved porous medium (Schwen et al., 2014)—**PDE**+**ODE**
Organism	*Pharmacokinetics* • Physiologically based whole-body PK, coupling GEMs to PK/PD—**FBA**+**ODE** (Bordbar et al., 2011; Krauss et al., 2012; Naik et al., 2014) • Lumped compartment PK models, e.g. acetaminophen liver model in PK/PD—**ODE** (Geenen et al., 2013) • With inter-individual differences (Krauss et al., 2013)—parameter adaption
Multi-Scale Integration	• Cellular metabolic network model integrated in whole-body PBPK model (Krauss et al., 2012)—**FBA**+**ODE** • Representative sinusoid: contains cells, contributes to organ, embedded in organism—**PDE**+**ODE** (Schwen et al., 2015) • Glucose regulation (sinusoidal models & PK/PD)—**ODE** (Ashworth W. B. et al., 2016) • Acetaminophen detoxification on multiple scales—**ODE** (Sluka et al., 2016)

*AB, Agent-based; CFD, computational fluid dynamics; FBA, flux-balance analysis; NAFLD, non-alcoholic fatty liver disease; ODE, ordinary differential equations; PDE, partial differential equations; PK/PD, pharmacokinetic/pharmacodynamic modeling*.

#### Models on the cellular scale

A comprehensive view of the various metabolic capabilities of the liver can be obtained via genome-scale metabolic models (GEMs) to analyze the flow of metabolites through hepatic metabolism based on steady state approaches. The most popular approach is Flux Balance Analysis (Orth et al., 2010). Multiple GEMs of the liver have been published (Gille et al., 2010; Jerby et al., 2010; Agren et al., 2014; Naik et al., 2014) and were applied to study central metabolic functions of the liver like the ${\text{NH}}_{4}^{+}$ detoxification (Gille et al., 2010), to predict metabolic fluxes across different hormonal and dietary conditions, or to simulate alterations as a consequence of gain or loss of function of single liver enzymes (Pagliarini et al., 2016). Such GEMs have proven useful as templates for the integration of omics data to understand the genotype-phenotype relationship in a mechanistic manner (Agren et al., 2014). In recent years, GEMs have been applied to stratify HCC patients (Björnson et al., 2015), to chart metabolic activity and functionality in non-alcoholic fatty liver disease (NAFLD) by integrating metabolic flux data and global transcriptomic data from human liver biopsies (Hyötyläinen et al., 2016), or to reveal alterations of metabolic pathways in NAFLD (Mardinoglu et al., 2014).

To date, GEMs have not been applied in the context of liver surgery, but coupling of omics data to analyse the global metabolic changes following liver resection and during regeneration could be an important next step.

An alternative approach is the use of kinetic pathway models based on ODEs. This approach focusses on specific metabolic functions by means of detailed mathematical description of the involved cellular processes and molecular players. Computational models of central liver functions have been developed, e.g., for the hepatic glucose homeostasis (König et al., 2012) providing insights into the switch of glucose pathways and the role of hormonal regulation. Additional examples are a minimal model of lipid metabolism in steatosis development (Schleicher et al., 2014) and a computational model of both hepatic glucose and lipid metabolism (Ashworth W. B. et al., 2016; Ashworth W. et al., 2016) yielding insight in the development of steatosis. Moreover, one possible mechanism involved in hepatic lipid deficiencies was elucidated by a detailed kinetic model of fatty acid beta-oxidation, in which an overload of substrate slowed down lipid degradation (van Eunen et al., 2013). Multiple pathways models for the detoxification of individual drugs have been published, e.g., for acetaminophen (Reddyhoff et al., 2015).

A more data-driven approach to metabolic function is to apply genome-wide omics data for phenomenological modeling of liver-related diseases. A large number of such studies exists, most of them aiming to identify key molecules, biological functions, and pathways relevant for the disease by differential omics analysis or via correlation-based networks and subsequent topological analysis. Omics-based models have been applied in the context of liver-related surgery, such as, e.g., in the analysis of pathobiochemical signatures of cholestatic liver disease after bile duct ligation in mice (Abshagen et al., 2015). Quantitative metabolomics was potentially useful to diagnose early graft dysfunction in liver transplantation (Serkova et al., 2007). Metabolomics data in orthotopic liver transplantation by consecutive liver biopsies revealed hundreds of significant metabolic differences between pre- and post-reperfusion grafts, among others increased urea production and bile acid synthesis (Hrydziuszko et al., 2010). Omics-based models will be an essential tool in understanding the alterations in liver functional capacity after resection and during regeneration.

#### Models on the sinusoidal and lobular scale

Kinetic pathway models, GEMs and omics approaches provide important information about metabolic functions and their alteration with disease. However, these approaches are limited, because they neither include tissue architecture nor perfusion, two important determinants of liver function especially in the context of liver surgery. Hepatic metabolism involves multiple spatial scales, ranging from metabolic pathways on the cellular scale via lobular zonation of metabolic properties and gradients of relevant compounds to metabolic heterogeneity on the organ level. Various multi-scale modeling approaches have been proposed (Diaz Ochoa et al., 2012; Kuepfer et al., 2012; Sluka et al., 2016) to represent the metabolism of the entire liver and especially the spatial heterogeneity of metabolic function on the lobule and organ scales.

One common approach of coupling metabolism to perfusion is treating the 1D porto-central axis of the sinusoid, consisting of a sinusoid surrounded by hepatocytes, as the repeating unit of the liver. Such ODE-based computational models were used to model the zonated damage and steatosis in NAFLD (Ashworth W. et al., 2016) or to analyze glucose homeostasis (Chalhoub et al., 2007; Ashworth W. B. et al., 2016), lipid metabolism (Schleicher et al., 2014, 2017), hepatic glucose and lipid metabolism (Chalhoub et al., 2007), the detoxification of xenobiotics like acetaminophen (Sluka et al., 2016), or effects of zonated damage on drug metabolism (Schwen et al., 2015, 2016). These sinusoidal unit models can be used as building blocks of whole-liver and whole-body models (for details, cf. Schwen et al., 2015; Sluka et al., 2016).

On the lobule-scale, metabolic pathway models have been integrated with agent-based models of perfusion and ammonia metabolism (Toepfer et al., 2007; Bartl et al., 2010, 2015; Schliess et al., 2014; Ghallab et al., 2016), contributing to a better understanding of how liver function depends on liver structure. In the agent-based approach, individual hepatocytes act as agents with intrinsic metabolism and behavior (like movement and proliferation). Such mathematical models have been applied to investigate the effect of liver damage on metabolic function after CCl_4_-induced necrosis (Schliess et al., 2014; Ghallab et al., 2016).

Alternatively, the liver lobule is modeled using homogenized continuum mechanical multiphase approaches, e.g., via the theory of porous media (Ehlers, 2002; Ricken et al., 2010, 2013, 2015; De Boer, 2012). Embedding a coupled system of ODEs in a porous medium model results in a spatio-temporal description of perfusion and metabolism. This approach was used to evaluate an anisotropic relation for the permeability of the liver lobule, the effect of outflow obstruction on liver remodeling and hepatic perfusion (Ricken et al., 2014), or the importance of vascular septa for homogeneous perfusion (Debbaut et al., 2014). Cellular glucose metabolism was coupled to the blood flow through a porous medium leading to an ODE/PDE (partial differential equations) model that helped to better understand glucose homeostasis on the lobule scale (Ricken et al., 2015).

An alternative approach for modeling perfusion is to apply Computational Fluid Dynamics (CFD) using detailed perfusion models in vessel geometries. CFD was applied to the liver to study blood flow in a segment of a lobule consisting of a resolved hepatic microvascular system (Rani et al., 2006). CFD was also used to simulate hemodynamic changes of the macro-circulation in the cirrhotic liver, a multi-scale computational model to simulate perfusion in the human liver on the organ and lobule scale (Peeters et al., 2015), and in liver cancer arterial perfusion models (Aramburu et al., 2016). A 3D multi-scale model of biliary fluid dynamics in the mouse liver lobule predicted drug-induced alterations of bile flow, and demonstrated that bile flow is driven by the osmotic effects of bile secretion and bile canaliculi contractility (Meyer et al., 2017). Until now the integration of metabolic models with CFD and porous medium models is very limited, and application in the context of liver surgery is missing.

#### Models on the whole-liver and whole-body scale

Sinusoid and lobule-scale models allow to represent the entire liver by applying appropriate scaling in a simplified way. Such models are based on the assumption that the organ does not contribute additional heterogeneity (e.g., in Sluka et al., 2016), or use multiple instances of such models “in parallel” to capture organ-scale heterogeneity (e.g., in Schwen et al., 2015). The organ scale has also been addressed directly via an ODE/PDE model of perfusion in the liver vessel tree and drug metabolization (Schwen et al., 2014). Tissue and whole-liver models allow to incorporate metabolic changes due to damage and resection by suitable adaptation of model parameters. With such approaches, effects of necrosis can be simulated on the lobule scale (Schliess et al., 2014) or changes in drug clearance can be predicted in steatotic livers (Schwen et al., 2014).

The liver in the context of the whole body is typically modeled using pharmacokinetic/pharmacodynamic (PK/PD) models (Jones and Rowland-Yeo, 2013) with a model spectrum ranging from detailed physiologically based models (Willmann et al., 2012) to strongly lumped models (Pilari and Huisinga, 2010). Many simplified models of various drugs being detoxified by the liver exist, often modeled via simple one-step reactions or a few reactions in the context of such PK/PD models (e.g., glutathione and acetaminophen metabolism; Geenen et al., 2013). For the liver, GEMs have been integrated into PK/PD models (Bordbar et al., 2011; Krauss et al., 2012; Naik et al., 2014) predicting, e.g., paracetamol clearance (Krauss et al., 2012). Examples of the coupling of sinusoidal metabolic models to PK/PD models are the analysis of glucose regulation (Ashworth W. B. et al., 2016) or acetaminophen detoxification (Sluka et al., 2016).

#### Perspective: metabolic models in computational liver surgery

Computational models of metabolic functions of the liver have been developed, many of them based on multi-scale approaches and integration of perfusion and tissue architecture. However, the application of such models to liver surgery, especially on how the metabolic function is changing after resection and subsequent regeneration, is still in its infancy. By coupling metabolic models to models capable of describing the effects of perfusion and morphology on liver function, a holistic understanding of changes after liver surgery on a local (tissue) and global (organ) scale could be achieved.

Surgical planning using model-based predictions of functional liver volumes could substantially improve clinical outcome. Importantly, computational models of hepatic metabolism could provide insights into the heterogenous distribution of metabolic liver functions like the heterogeneity of fat in NAFLD and its consequences for the regional functional capacity. Multi-scale metabolic models of NAFLD/steatosis would allow to calculate hepatic functional capacity based on given fat content, tissue properties like stiffness and elasticity, and perfusion. Thereby, they would provide important insights into surgical planning. Multi-scale computational models of metabolic functions may also improve evaluation of quantitative liver function tests, like galactose elimination capacity or LiMAx. Integrated with surgical planning tools, computational models of such liver function tests could provide a more accurate prediction of metabolic function after resection and during regeneration.

Integrating omics data with metabolic models for predicting changes after liver surgery seems a promising future direction. Personalizing generic models based on individual omics data, a personalized prediction of metabolic liver function and its alteration after resection could be achieved. This personalization as well as the stratification of patients into subgroups has already been demonstrated (Björnson et al., 2015; Hyötyläinen et al., 2016). The use of omics data, however, is not yet part of clinical routine, but could be important for the prediction of the remnant liver function and thereby surgical planning in the future. For individual function predictions, computational models could be parametrized with a subset of omics data relevant for the respective model.

### Regeneration

The liver is capable of regenerating both volume and function after physical damage induced by medical interventions. This includes damage at the lobule scale induced by intoxication with CCl_4_ (Weber et al., 2003) or damage at the organ scale due to surgical interventions (Riehle et al., 2011), as well as spatial and functional graft adaptation after transplantation (Taki-Eldin et al., 2012). Once the liver is damaged, loss of hepatic mass leads to an increase in portal blood flow per unit mass followed by metabolic overload in the remaining tissue and an increase in diverse signaling molecules including IL-6, TNFα, HGF, and EGF (Michalopoulos, 2010). These signaling molecules, as well as Hedgehog signaling (Matz-Soja, 2017), jointly orchestrate the tightly controlled process of hepatocellular proliferation. This process is composed of three phases: priming (initiation), proliferation, and termination (Fausto, 2000). Mathematical modeling of the involved biological processes in the different phases of regeneration has the potential to aid in understanding the underlying molecular mechanisms.

In this section, we review existing phenomenological models of biological tissue growth, followed by mechanistic models, which include relations and interactions between the involved biological processes specifically during liver regeneration. A summary of selected models available to address regenerative processes in the liver, which might be relevant for surgical planning, is given in Table 3.

**Table 3:** Selection of existing computational models addressing regeneration processes with potential relevance for surgical planning, sorted according to spatial scale (*cell to organism*).

**Scale**	**Modeling Approaches**
Cell	*Proliferation and its regulation* Identification of molecular mechanisms (Zhou et al., 2014)—**correlation network**
Lobule	*Growth and remodeling* Continuum mechanical models of soft tissue—**multiphase-PDE** (Ricken and Bluhm, 2009) Mixture theory—**multiphase-PDE** (Hum2002, BenGor2005, AmbPetRicStyCia2016) Growth of biological tissues—**multiphase-PDE** (Ateshian and Ricken, 2010) Onephasic—**multiphase-PDE** (Menzel and Kuhl, 2012) Biphasic—**multiphase-PDE** (Ricken et al., 2015) Triphasic—**multiphase-PDE** (Ricken et al., 2007; Ricken and Bluhm, 2010; Waschinsky et al., 2016)
	*Regulation of regeneration* After CCl4 intoxication—**agent-based**+**ODE** (Hoehme et al., 2010) By perfusion or metabolic load in model sinusoid (1D hepatocyte layer)—**IPS** + **ODE** (Hohmann et al., 2014)
Organ	*Tissue growth* Continuum mechanics—**PDE** (Garikipati et al., 2004)
	*Volume recovery* Liver size—**ODE** (Shestopaloff and Sbalzarini, 2014) Liver size taking into account extrahepatic parameters (such as BMI)—**ODE** (Yamamoto et al., 2016)
	*Regulation of growth* Molecular species and number/growth of liver cells—**ODE** (Furchtgott et al., 2009; Periwal et al., 2014; Cook et al., 2015) Role of bone marrow cell migration in damaged tissue—**ODE** (Pedone et al., 2017)
Organism	(none)
Multi-Scale Integration	• Cells in lobule—**AB**+**ODE** (Hoehme et al., 2010) • Cells at sinusoid—**IPS**+**ODE** (Hohmann et al., 2014)

*AB, Agent-based; IPS, interacting particle system; ODE, ordinary differential equations; PDE, partial differential equations*.

#### Phenomenological models of liver volume regeneration

Different types of models have been developed to simulate biological growth (see, e.g., the reviews Ambrosi et al., 2011; Jones and Chapman, 2012) and its regulation (Chara et al., 2014), in particular continuum mechanics models of growth (Skalak et al., 1982; Lubarda and Hoger, 2002), for soft tissues (Rodriguez et al., 1994; Garikipati et al., 2004; Himpel et al., 2005), or tumors (Greenspan, 1976). Such models are able to calculate the mechanically induced volumetric growth of tissue without explicitly resolving the underlying biological structures and mechanisms.

A model for volumetric growth of organs including quantitative characteristics and geometric shape of the liver (Shestopaloff and Sbalzarini, 2014) was used to quantitatively estimate patient-specific optimal size and shape of liver transplants. Volume recovery computed from 3D image data, such as shown in Haga et al. (2008), is a typical way of quantifying regeneration and can be used to either calibrate or validate the models and their predictions.

A model predicting postoperative liver volume regeneration from individual quantitative clinical data was recently developed (Yamamoto et al., 2016). This phenomenological model predicted, whether liver size would recover or remain irreversibly reduced, based on preoperative physiological and functional parameters as well as parameters of the surgical procedure.

#### Mechanistic models of liver volume regeneration

##### Temporal models

Several studies aimed to mathematically model liver regeneration based on known interactions between regeneration-associated biological processes and representative molecules. These models are based on ODEs or delayed differential equations, and thus focus on the temporal scale of the process of regeneration without resolving spatial processes, in particular assuming spatial homogeneity. A model reflecting the interplay of cytokines and growth factors involved in initiating and terminating liver regeneration (Furchtgott et al., 2009) was used to derive different hypotheses for the improvement of liver regeneration. This model was later transferred to modeling of human liver regeneration in living liver transplant donors (Periwal et al., 2014). Further extensions of the model by Furchtgott et al. (2009) was used to emphasize the role of bone marrow cell migration into the liver after resection in mice (Pedone et al., 2017), and to integrate cell growth and its regulation, also in case of model diseases (Cook et al., 2015). The aforementioned models assume that the metabolic overload induces regeneration. However, studies also hypothesize that the increased portal flow per mass unit initiates the process of liver regeneration. The two hypotheses were assessed by comparison of two models reflecting liver regeneration as a consequence of hemodynamic changes or the metabolic overload (Hohmann et al., 2014).

##### Spatio-temporal models

A number of studies also focused on including spatial properties of liver regeneration. Already half a century ago, an ODE-based model for cells at the sinusoidal scale was presented (Sendov and Tsanev, 1968), involving proteins, ribosomes, DNA, and mechanisms predicting cellular death and division. Such models could be used to identify hepatocyte-specific triggers when applying general cell cycle models (see e.g., Kriete et al., 2014). A mechanistic model at the cellular and lobular scale for liver regeneration after CCl_4_-induced damage at the lobule scale in mice was used to show that not only hepatocyte proliferation but also coordinated cell orientation as well as cell polarity are critical aspects ensuring restoration of the lobular micro-architecture (Höhme et al., 2007; Hoehme et al., 2010).

One common approach for spatio-temporal continuum models of regeneration is using mixture theory (multiphasic approaches) embedded into a biomechanical framework. This allows the integration of underlying biological mechanisms, see among others (Humphrey, 2003; Amar and Goriely, 2005; Ambrosi et al., 2017). The field of growth and remodeling in biomechanics is covered by one-phasic (Menzel and Kuhl, 2012) as well as bi-phasic (Ricken and Bluhm, 2009) approaches. Remodeling processes are presented in Ricken et al. (2010), where a mechanical biphasic model was developed and the effect of outflow obstruction on liver remodeling and hepatic perfusion was studied. Dealing with coupled solid-fluid interaction, a mixture framework using the finite element method was presented (Ricken et al., 2015). This approach allows calculating tissue growth depending on nutrient supply, e.g., diet high of free fatty acids (Waschinsky et al., 2016).

##### Network models

Furthermore, omics-based network models are commonly used for the initial identification of genes and proteins involved in liver regeneration and are thus used to identify key-molecules to be considered in mechanistic models. However, few studies have employed mathematical modeling based on genome-wide transcriptomics data for the identification of liver regeneration-associated molecular mechanisms and biological pathways. A correlation-based model was inferred from genome-wide transcriptomics data for the identification of molecular mechanisms underlying regeneration induced by partial hepatectomy (Zhou et al., 2014). This identified de-regulation of several genes associated with hepatocyte proliferation, inflammation, and DNA replication processes.

#### Models of liver function recovery

Only few models of recovery of liver function have been reported, most of them being phenomenological. Liver function (in particular the lack thereof) has mostly been addressed in terms of postoperative liver failure. Well-known risk factors for postoperative liver failure are, e.g., preexisting disease, age, nutrition (Hammond et al., 2011). The risk of liver failure can be predicted partly by preoperative tests and risk-defined score models (Clavien et al., 2007) and additionally by postoperative parameters (Yamanaka et al., 1984).

A mechanistic model (Schliess et al., 2014; Ghallab et al., 2016) of function recovery on the tissue scale deals with the recovery of ammonia detoxification and amino acid metabolism during regeneration after CCl_4_-induced pericentral necrosis. This model included two selected aspects of liver function and regeneration from damage pattern clearly different from those encountered in surgery, but could be used as a starting point for bridging the cellular and organ scale in regeneration modeling in computational liver surgery.

#### Perspective: regeneration models in computational liver surgery

To our knowledge, currently no mechanistic mathematical model addresses liver regeneration after hepatic surgery. Future models supporting the prediction of regeneration could be integrated in surgical risk assessment and help preventing postoperative complications. The existing predictions of liver failure could be extended to predicting the recovery of liver function based on more advanced and more mechanistic models. The tissue-scale function recovery model (Schliess et al., 2014; Ghallab et al., 2016) could form the basis for a model describing changes in lobular architecture and its impact on more generic function recovery after resection. The main challenge for modeling recovery of liver function is to link tissue regeneration to metabolism, as already described in the previous subsection. Moreover, correlation of volume and function recovery for different diseases (Yamanaka et al., 1993) could be used for phenomenological models of functional recovery.

## Vision: systems surgery of the liver

Future integrated models of liver metabolism and regeneration should provide function-based risk assessment. Such models need to be accessible via a usable tool for surgery planning. To achieve a more accurate and comprehensive prediction of the functional capacity for Systems Surgery, several medical and computational challenges have to be resolved (Belghiti, 2016).

These challenges involve (a) precise determination of the preoperative state and functional capacity of the liver, taking preexisting disease into account (model input data), (b) estimation of the extent of surgical damage inflicted on the liver during the resection, and (c) prediction of the impact of hepatic tissue loss and surgical damage on the functional capacity of the (diseased) future liver remnant and its recovery process (model output data).

### Integrated planning tool for liver resection

**Figure 4:**
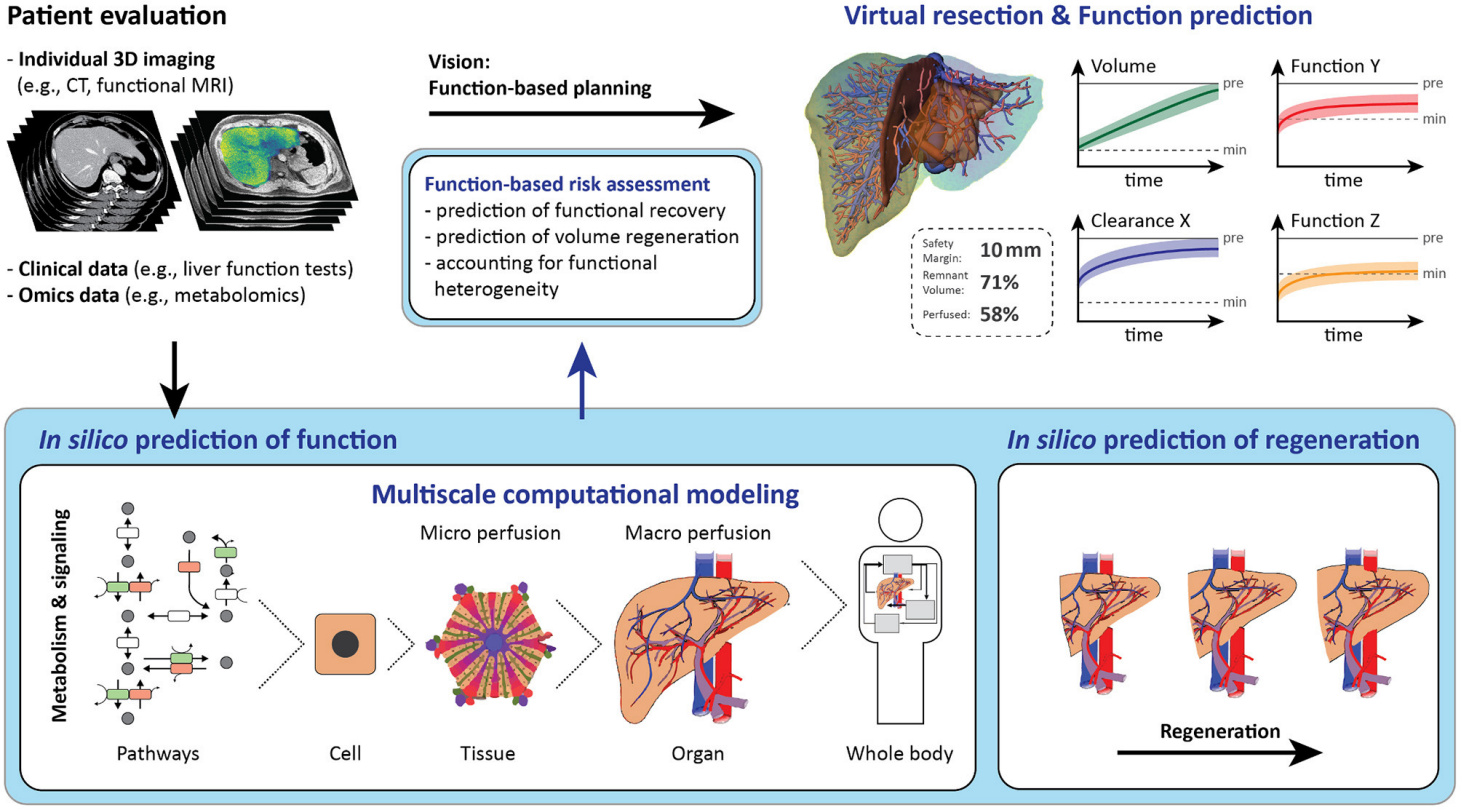
Vision of future liver surgical planning tools. Surgical planning tools of the future will improve risk prediction by accounting for the functional heterogeneity of the healthy and diseased liver and by providing predictions of the functional capacity of the future remnant liver. Multi-scale computational models of the liver will provide the required *in silico* prediction of function and regeneration (blue box). Key information for surgical planning are time-resolved functional recovery curves, e.g., how clearance of certain substances is affected and recovers after resection. Suitable computational models have to be integrated and validated based on animal models and clinical data (for an overview over computational models of the liver applicable in the context of surgical planning see Tables 1–3). The input data for such function-based risk assessment includes in addition to the assessment of liver geometry, also the spatially resolved assessment of hepatic perfusion and hepatic function as well as clinical data, e.g., quantitative dynamical liver function tests, and information about existing liver disease. Additional output of the future surgical planning tool includes prediction of selected functions after resection, (e.g., hepatic perfusion, metabolic parameters) and their recovery in respect to variation of resection surface and safety margins. CT image stack adapted from (Figure 1B in Chung et al., 2013), image license: CC-BY (https://creativecommons.org/licenses/by/3.0/).

Future surgical planning software should include a workflow for function-based risk assessment. Input data data comprise in addition to the liver anatomical architecture also spatially resolved data assessing hepatic perfusion and function as well as clinical data, e.g., quantitative dynamical liver function tests, and information about existing liver disease as summarized graphically in Figure 4. Multi-scale computational models of the liver based on animal models and clinical data will enable to predict *in silico* function and regeneration after resection in respect to variation of resection surface and safety margins.

Functional predictive models need to be integrated into the existing individual 3D planning of the liver representing the vascular structure of the liver and the location of the tumor.

Such an integrated planning tool will improve individualized risk prediction for hepatic surgery and provide important information about the expected liver function after surgical intervention and during subsequent liver regeneration. This tool will ultimately support surgeons in their decision about a patient's operability and the choice of a suitable intervention, but will also make them aware of possible postoperative complications allowing therapy adjustments after resection.

The integrated tool will support risk assessment depending on preexisting liver disease and damage of the liver. This requires integrating underlying pathophysiologic conditions and preexisting risk states on an individual basis. For example, steatosis and other chronic liver diseases (such as cirrhosis already impairing liver function) substantially impact the function of the future liver remnant and its regeneration, and hence increase the risk of postoperative complications and liver failure. Surgeons and patients will benefit from more comprehensive risk predictions taking functional aspects into account without the need of own expertise in multi-scale computational modeling in the implementation.

### Future developments

Further development of such an integrated liver model can be envisioned to obtain better disease- or cohort-specific predictions and to enhance the prognostic power for the individual patient.

Reaching better disease- or cohort -specific predictions would call for including further cohort-specific data to tune the integrated model according to the specific aspect in question. Doing so will contribute to getting a better insight into disease progression and curation. However, this will require to generate considerably more animal experimental data of the specific disease and of course to collect a substantial amount of additional cohort-specific clinical data.

Such data are needed to generate probabilistic disease models, which have to be integrated into the proposed “liver resection and regeneration” model.

Enhancing the prognostic power for the individual patient could be achieved by extending the knowledge-based selection of relevant patient-specific pre-, peri-, and postoperative data considered to be relevant. Additional input data regarding the activity and severity of the complicating liver disease as well as data regarding the general patient condition (e.g., cardiovascular condition) appears extremely useful for this purpose. Similarly, additional outcome data would be necessary, requiring an detailed follow-up of the patient to collect data regarding extra-hepatic surgical and general complications [e.g., abscess formation, postoperative infections and grade their severity (Clavien-Dindo classification)] and reflecting the recovery of the patient's general condition (e.g., days in ICU and in hospital). However, increasing the number of entry variables would call for a higher number of outcome observations.

Alternatively, this could also be achieved using a “big data” approach by focussing on creating an interface with the currently used hospital information systems to have access to all patients and all patient-specific information. Following this approach, a rather large number of patients would be needed to reflect the high data variability as presented in true patient cohorts.

### Medical challenges

Determining the preoperative state of the liver and the expected alterations after surgery must be improved to optimize surgical planning and reduce the probability of postoperative liver failure. This involves several challenges.

#### Improving preoperative diagnostics

Here, the key point is to improve spatial resolution, which will benefit the assessment of morphological and structural alterations due to the underlying preexisting hepatic disease, the assessment of hepatic perfusion, and most importantly, the quantitative assessment of hepatic function.

#### Identifying prognostically relevant aspects of hepatic function and their spatially resolved assessment

Identifying meaningful and relevant diagnostic assays from the multitude of available assays is a major challenge. These assays should be non-invasive and serve as a basis for valid predictions regarding surgical complications, surgical outcome, and changes in liver functions following liver surgery.

#### Estimating surgically induced damage

It is not sufficient to only quantify the loss of liver volume due to tissue removal, but also necessary to quantify the volume of liver tissue at risk due to alterations of hepatic perfusion. The key challenge is to estimate the loss of functional tissue with respect to the extent of resection, the resection surface, and the resection technique. In addition, preexisting global liver diseases impair hepatic function in a spatially heterogenous way (cf. the section “Hepatic Diseases”), which has to be taken into account during the surgical planning phase.

#### Predicting postoperative function of the liver remnant

The functional capacity of the remnant liver should be predicted based on the preoperative disease state and the predicted loss of liver tissue and liver function by resection.

### Modeling challenges

Building a comprehensive model for the prediction of the hepatic functional capacity after resection faces many challenges.

#### Identifying, understanding, and modeling relevant processes in liver surgery

A prerequisite for building a comprehensive model of functional prediction is the availability of high quality models reflecting those aspects that are important for liver surgery, such as liver function depending on perfusion, liver volume regeneration in case of preexisting damage, or recovery of hepatic metabolic function after resection. The key processes and mechanisms of all these aspects must be understood in sufficient detail and transferred to a suitable mathematical formalism. Part of the challenge is to extend compatible model components and to develop interfaces to bring these building blocks together.

#### Improving data availability and quality for computational models

Besides understanding the processes, further key steps for model building are parameterizing and subsequently validating parametrized models. A key requirement for these steps is the availability of high-quality experimental and clinical data.

Many existing studies have only looked at a single aspect of liver surgery, such as regeneration, liver function, or changes in perfusion. Assembling data from different sources is difficult, since the experimental and clinical conditions are in general vastly different. A multitude of experimental resection studies has been performed in rodents under controlled conditions and with various liver diseases, but using a variety of experimental conditions and read-out parameters. Consequently, comparability is often limited and data integration into a single model questionable. A similar problem is the extrapolation from clinical data measured in one cohort to another cohort with different characteristics, e.g., data from young subjects to old subjects. For similar reasons, translation of results from animal studies to the human situation is even more challenging.

One recurring problem is the quality of experimental and clinical data, e.g., inaccurate or high-variance data, with model predictions strongly depending on the accuracy of the measured patient-specific data. The aforementioned issues with experimental data require new comprehensive and targeted data sets to ensure all needed input data were generated under the same conditions. One key aspect is to perform targeted experiments and studies to collect the information for model parameterization and validation. Alternatively (or in addition), analysis of the effects of the underlying datasets on model predictions and quantification of the resulting uncertainties in the predictions must be performed in order to analyze sensitivity.

#### Integrating data with computational models

One major challenge is the integration of different types of data (e.g., concentrations, tension, elasticity, image data, omics data, etc.) and to handle the heterogeneity within similar datasets (e.g., from different laboratories, different readers) with computational models. Standardization of data formats and models for simple and reproducible integration of the different data sets into the models is important (König et al., 2016). Especially with the perspective of routine application of such models in Systems Surgery of the liver, standardization of models and experimental data sets will be a major challenge and facilitator.

#### Developing large multi-scale models

Multi-scale models and models coupling distinct modeling approaches are often not easy to compute. Reasons are that such large computational models require substantial computational resources (e.g., agent-based, porous media, CFD), and that coupling of different modeling is often not supported in simulation software and difficult to implement. Multi-scale computational modeling requires connecting models via clearly defined interfaces between the different scales and sub-models. General challenges of computational modeling like parameter fitting/overfitting, model selection, parameter selection, or parameter identifiability are also major challenges in multi-scale models, often aggravated due to the large number of parameters in models spanning multiple scales.

#### Performing model reduction

Often, model reduction is necessary for efficient model simulation (e.g., integration of a system of ODEs for metabolism in a meso- or macroscale model of whole-liver perfusion) and reduction of the parameter space for analysis. The overall goal is to reduce complexity without compromising the aspects relevant for the question at hand. Different approaches of model reduction have been applied in the field of liver simulations, e.g., representative sinusoids (Schwen et al., 2015), method of proper orthogonal decomposition (Fink and Ehlers, 2015), or the use of an energy function (Holzapfel et al., 2000; Humphrey, 2003; Balzani et al., 2006).

#### Improving model quality and validating predictions

Further important challenges are the evaluation of model quality and validation of model predictions, which are two requirements for application of such models in surgical support systems. Validation of models for Systems Surgery of the liver will require prospective clinical trials, which compare the model predictions of liver function after resection and during regeneration with clinical trial data. In the surgical setting, the availability of postoperative data (invasive methods for data measurements are not feasible) limits model validation, so this will need to be done mostly in animal models.

#### Quantifying uncertainty and robustness

Important questions to be answered in the context of model validation are (a) What is the uncertainty in input data and model parameters? and (b) How sensitive is the overall system? Together, this can quantify how robust model predictions are against uncertainty in the generic model parameters and individualized input data. There are various sources of uncertainty, e.g., direct or indirect measurement of biochemical and biophysical parameters, clinically measured physiological and systemic functional parameters, limited resolution, and noise in imaging. An analytic assessment of the sensitivity is only feasible for sub-models of limited complexity. Quantifying the robustness of an integrated multi-scale model will require thorough parameter studies to quantify the sensitivity against uncertainty in individual parameters and, e.g., Monte-Carlo simulations to determine confidence ranges of model predictions under combined parameter uncertainty.

### Implementation challenges

Addressing these clinical and modeling challenges to achieve such model-assisted risk predictions requires a truly multidisciplinary approach involving basic and applied, clinical and computational scientists and engineers. On the one hand, anatomical and physiological phenomena, as well as clinical diagnostic and surgical procedures, need to be accurately described and translated to suitable, improved or novel, computational models. On the other hand, such models must be made available in the form of interactive and user-friendly software and thus usable not only by domain experts in systems biology. Practical usability requires user interactivity, easy and quick handling, automation, minimum of editing, and expert input in the final usage, model adaptation to work on standard workstations available in the clinics, etc. Moreover, interfaces have to be developed, which allow integration of computational models with widely used hospital information systems, e.g., using patient data for the personalization of models and adding model-based risk evaluation to electronic patient records.

## Conclusion

Already today, patients benefit from computational support in the planning of liver resections. This is, however, limited to an assessment of remnant liver volume and taking into account a number of risk factors for postoperative liver failure. A prediction of liver function recovery is currently not included, but would be particularly useful in case of preexisting liver disease.

Basic biological processes involved in liver metabolism, disease, and regeneration are well-understood, and various computational models for these aspects are available. However, no comprehensive model integrating all these effects on different scales has been presented yet.

With increasing knowledge of disease mechanisms, availability of experimental and clinical data as input for model-based predictions, and expertise in design and integration of computational models, the next logical step is to develop a comprehensive model for predicting liver function and regeneration. This type of outcome prediction will be an indispensable part of a strategy for a patient-tailored optimization of intervention and therapy after liver surgery.

## Author contributions

All authors listed have made a substantial, direct and intellectual contribution to the work, and approved it for publication.

### Conflict of interest statement

The authors declare that the research was conducted in the absence of any commercial or financial relationships that could be construed as a potential conflict of interest.
